# Targeted Surgical Repair of a Symptomatic Hepatic Artery Aneurysm: Case Insights and Outcomes

**DOI:** 10.1155/cris/5774622

**Published:** 2026-01-05

**Authors:** Alessandro Robaldo, Francesca Mariani, Alessandra Cristaudi, Giorgio Prouse, Pietro Majno-Hurst, Luca Giovannacci

**Affiliations:** ^1^ Department of Vascular Surgery and Angiology, Ospedale Regionale di Lugano, Ente Ospedaliero Cantonale, Lugano, Switzerland, eoc.ch; ^2^ Department of Surgery, Ospedale Regionale di Lugano, Ente Ospedaliero Cantonale, Lugano, Switzerland, eoc.ch

## Abstract

**Introduction:**

Hepatic artery aneurysms (HAAs) are rare but significant vascular lesions associated with high mortality due to rupture, particularly in symptomatic cases. This report highlights the clinical importance of timely intervention and presents a case of surgical management of a true HAA.

**Case Presentation:**

We present a 58‐year‐old man with a history of Crohn’s disease who exhibited acute right upper quadrant and epigastric pain. Initial evaluation, including CT angiography (CTA), revealed a 24‐mm fusiform HAA involving the common hepatic artery. Despite transient relief of pain with analgesics, surgical intervention was deemed necessary due to the aneurysm’s size and risk of rupture. The patient underwent an aneurysmectomy with reconstruction using an inverted great saphenous vein graft through a bilateral subcostal incision. The surgical procedure was completed without intraoperative complications, successfully excising the aneurysm and restoring blood flow via the graft. The patient experienced a complex postoperative course, including a sudden episode of bleeding from the left gastric artery, which was effectively managed with endovascular coil embolization. Follow‐up imaging at 12 months showed no residual aneurysm and confirmed graft patency, along with favorable perfusion of the intrahepatic arteries.

**Conclusions:**

This case illustrates that surgical repair can be a safe and effective treatment for HAAs when endovascular options are not feasible. It underscores the necessity of personalized management strategies based on individual patient characteristics and specific aneurysm features. Further studies are required to optimize treatment protocols for HAAs.

## 1. Introduction

Hepatic artery aneurysms (HAAs) are rare but require prompt management due to their high propensity for rupture and the significant associated mortality, particularly in symptomatic cases. These lesions represent the second most frequently reported type of visceral artery aneurysm. All segments of the hepatic artery are susceptible, with the majority (75%–80%) being solitary and occurring extrahepatically. Although information regarding the best treatment options is limited and current guidelines are based on a low level of evidence, medical, surgical, and endovascular treatments have been described. The endovascular approach is increasingly recommended as the preferred solution, while open repair remains a valuable option [1]. This work is reported in accordance with the SCARE 2023 guideline [2].

## 2. Case Presentation

A 58‐year‐old white male with a history of Crohn’s disease, prior hepatitis B infection, metabolic syndrome, nonalcoholic fatty liver disease, and major abdominal surgery (laparotomic sigmoidectomy for diverticulitis complicated by anastomotic insufficiency and peritonitis involving all four quadrants, 8 weeks post‐procedure) was urgently referred to our department due to sudden, severe right upper quadrant and epigastric pain, without any clinical signs of infection or other identifiable pathologies to explain the symptoms. Laboratory tests returned negative results for both liver function and inflammatory markers. A CTA revealed a 24‐mm fusiform hepatic aneurysm extending from the origin of the common hepatic artery to the trifurcation of the proper hepatic artery. Additional findings included a 12‐mm dissected dilation of the celiac trunk and a sub‐occlusive stenosis at the origin of the common hepatic artery (Figure 1). There was no reported family history of arterial aneurysms, and no other vascular abnormalities were found during the physical examination. An esophago‐gastro‐duodenoscopy was conducted to exclude alternative etiologies for the epigastric pain. After 24 h, the pain subsided with adequate analgesic therapy. Despite this favorable clinical evolution, a collegial discussion led to the decision to treat the lesion due to its size and risk of rupture. Blood cultures and preoperative cardiac testing were negative. After providing a comprehensive explanation of treatment options and associated risks, written informed consent was obtained from the patient. The therapeutic strategy was defined through a structured stepwise evaluation. Angiographic assessment first confirmed the aneurysm morphology, extent, and vascular anatomy. Endovascular repair was considered but ultimately excluded because of the absence of a suitable distal landing zone, marked arterial tortuosity, and the potential risk of hepatic ischemia related to gastroduodenal artery coverage. Embolization or ligation was also ruled out, as the collateral circulation was insufficient and the risk of hepatic infarction was deemed unacceptably high. Consequently, open surgical repair was selected as the definitive treatment, with reconstruction of the hepatic artery using an autologous reversed saphenous vein graft to ensure preservation of intrahepatic perfusion. Using a bilateral subcostal incision, careful surgical exposure was achieved to visualize the celiac trunk and its branches, as well as the gastroduodenal artery and the proper hepatic artery along with its right, middle, and left terminal branches. An aneurysmectomy was performed, and the hepatic artery was reconstructed using a reversed great saphenous vein graft, which was anastomosed end‐to‐end to the stump of the celiac trunk and distally end‐to‐end to the distal part of the proper hepatic artery (Figure 2). The clamping time was 29 min. No signs of ischemia were observed upon completion of the procedure. Intraoperative duplex scanning confirmed both patency and a good flow signal in the intrahepatic arteries. No intraoperative complications occurred. On postoperative Day 7, the course was complicated by sudden anemia due to active bleeding from the left gastric artery, as detected by CTA. This was successfully managed via endovascular coil embolization. The remaining postoperative course was uneventful, and the patient was discharged 15 days after surgery in good condition, without signs of inflammatory syndrome, hepatic dysfunction, renal failure, or recurrent abdominal pain. The CTA at 1 month revealed no residual aneurysm, although a suspected stenosis of the proximal anastomosis was noted, which was not confirmed on follow‐up angiography, and no pressure gradient was recorded across the anastomosis (Figure 3A,B) Clinical follow‐up with duplex and CTA at 3 and 12 months showed a patent graft, stable moderate proximal anastomotic stenosis without hemodynamic significance, adequate intrahepatic perfusion, no residual aneurysm, and no complications (Figure 3C).

Figure 1CTA scan shows the hepatic aneurysm and dilation of the celiac trunk. (A) Axial image demonstrating the 22 mm hepatic aneurysm. (B) Sagittal image demonstrating the dilation of the celiac trunk with a dissecting flap. (C) 3D reconstruction shows patency of distal branches and the gastroduodenal artery, without a suitable landing zone for stenting or safe embolization.

**Figure:**
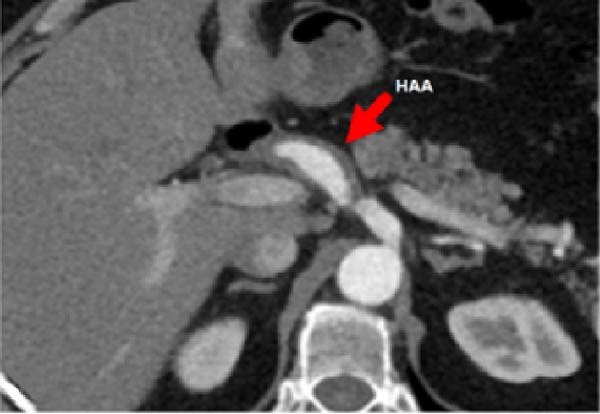
(A)

**Figure:**
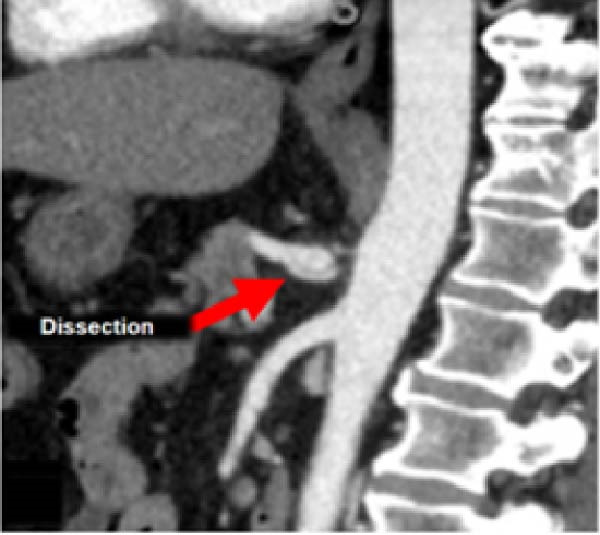
(B)

**Figure:**
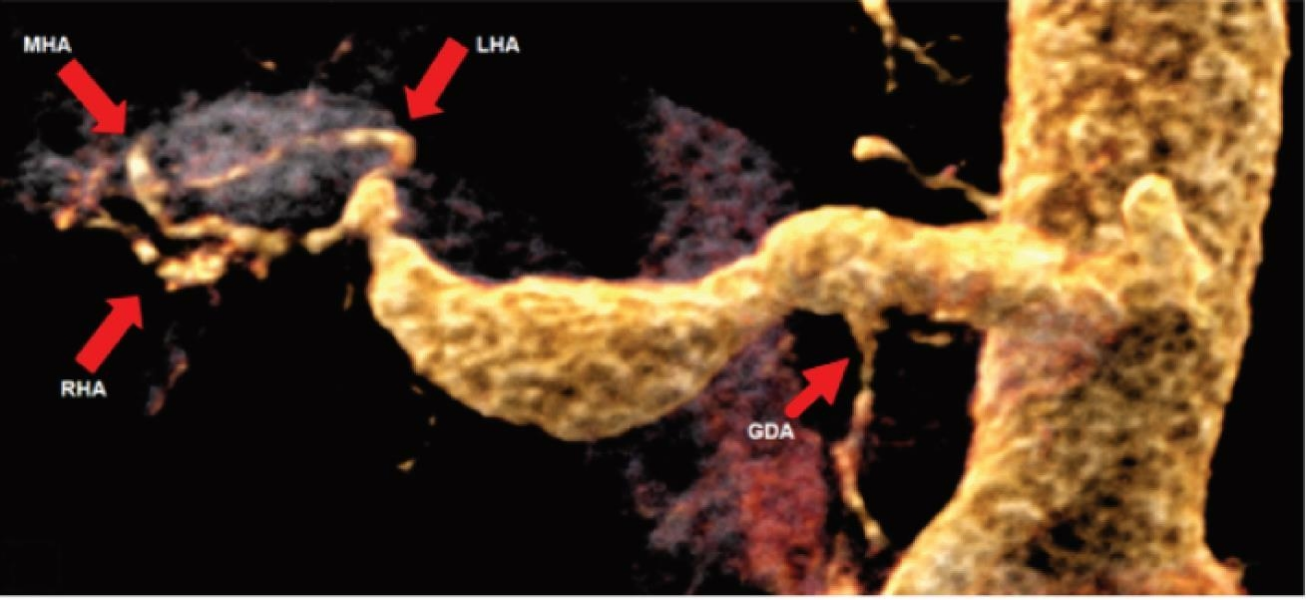
(C)

Figure 2Intraoperative images. (A) The hepatic artery aneurysm with its branches: left proper hepatic artery (1), middle proper hepatic artery (2), right proper hepatic artery (3), accessory pancreatico‐duodenal branch (4), splenic artery (5), and left gastric artery (6). (B) The great saphenous graft anastomosed proximally to the celiac trunk and distally to the proper hepatic artery.

**Figure:**
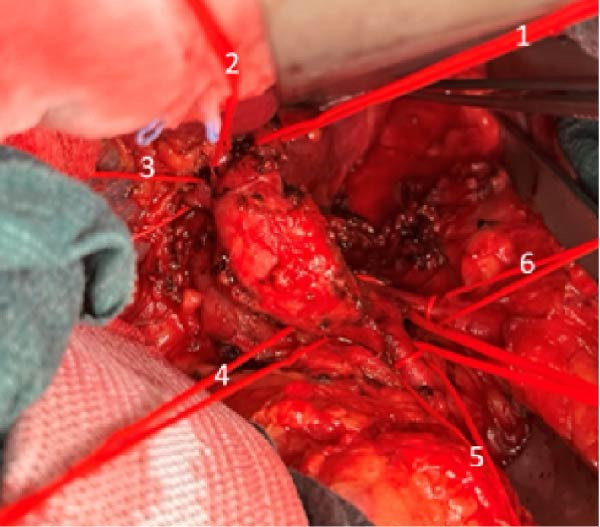
(A)

**Figure:**
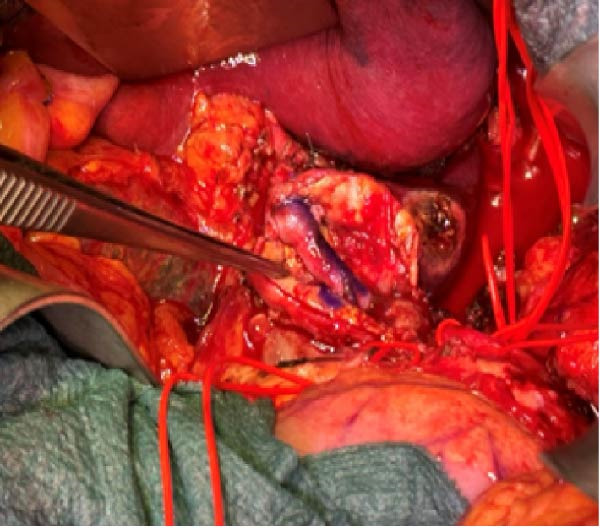
(B)

Figure 3Follow‐up imaging show: (A) CTA scan at 1 month showing the patency of the venous hepatic bypass. (B) Angiography showing the patency of the bypass without anastomotic stenosis and the outcome of the left gastric artery embolization. (C) 3D reconstruction at 12 months shows patency of its branches.

**Figure:**
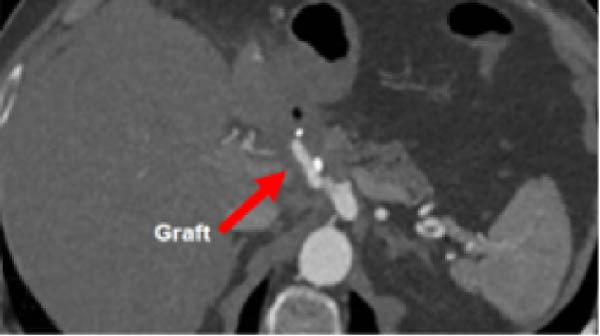
(A)

**Figure:**
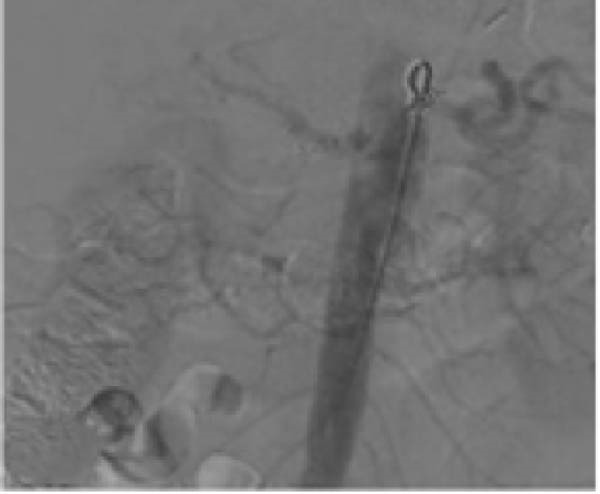
(B)

**Figure:**
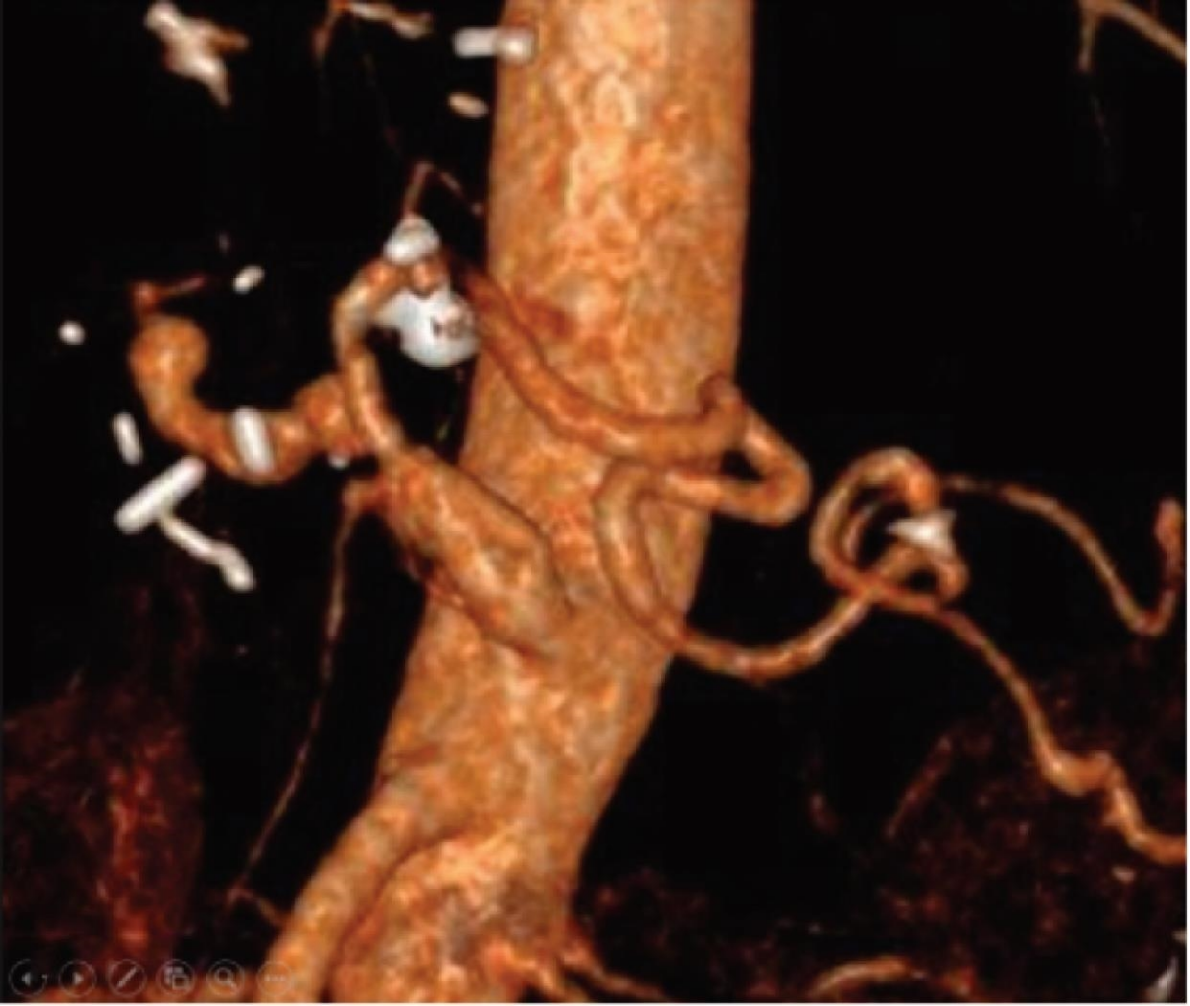
(C)

## 3. Discussion

HAAs represent 20% of all visceral aneurysms and can be potentially catastrophic, with a death rate as high as 40% following spontaneous rupture [3]. The most common causes of HAAs are atherosclerosis and medial–intimal degeneration. Less frequently, infections, fibromuscular dysplasia, perilesional inflammation, trauma, or iatrogenic factors are implicated. Systemic medium‐vessel vasculitides, including those associated with hepatitis B virus infection, have become markedly less common due to widespread vaccination and effective antiviral therapies [4]. In this case, the absence of active HBV infection or clinical/laboratory signs of vasculitis makes a direct causal link unlikely. Although recent major abdominal surgery is a known risk factor for pseudoaneurysm formation from iatrogenic or traumatic injury, true aneurysms involve all three vessel wall layers and are not typically surgery‐related [4, 5]. Autoimmune and inflammatory disorders (e.g., systemic lupus erythematosus, Behçet’s disease) are rarely linked to visceral aneurysms; HAAs have not shown a consistent association, and only isolated cases have been described in Crohn’s disease [1].

We hypothesize that the etiopathogenesis in this patient most likely reflects an atherosclerotic rather than infectious, autoimmune, or postsurgical origin, consistent with clinical findings and current literature. HAAs often present with nonspecific symptoms that depend on their size. Small lesions may be asymptomatic initially, but their natural progression increases the risk of rupture and mortality. Symptoms can include right upper quadrant and epigastric pain, biliary tract obstruction, and, in 30% of cases, the classic triad of Quinke’s—obstructive jaundice, abdominal pain, and hemobilia [6]. The increased use of imaging scans for various purposes has led to more frequent incidental detection. Prompt diagnosis and treatment are crucial to prevent rupture into the abdominal cavity, bile duct, or bowel. While abdominal ultrasound can be used for initial evaluation or follow‐up, CTA is considered the most accurate diagnostic tool, with 100% sensitivity and specificity. Furthermore, the exam provides detailed information about vascular anatomy and aids in planning appropriate treatment strategies. Regarding indications for invasive intervention, American guidelines recommend the repair of asymptomatic HAAs with a diameter >20 mm, while European guidelines recommend treatment for those >30 mm. Both guidelines advocate for treating symptomatic patients and cases of pseudoaneurysms, regardless of size. Nonoperative treatment, which includes antithrombotic therapy and regular follow‐up, is considered for selected asymptomatic patients with significant comorbidities and aneurysms <5.0 cm [1, 7]. However, it’s important to note that current evidence is limited, primarily derived from small retrospective observational studies, and challenges remain in defining precise repair indications. Some authors recently reported 10 cases of ruptured HAAs, three of which had an aneurysm diameter <20 mm [8]. The choice of therapeutic intervention is influenced by factors such as the size, etiology, and location of the aneurysm, as well as the patient’s age and comorbidities. In patients with systemic inflammatory disorders, concerns may arise regarding vessel wall fragility and impaired healing, potentially increasing the risk of postoperative bleeding or stenosis. Such complications are well documented in vasculitides, where aneurysm recurrence and graft failure are more common [9]. By contrast, Crohn’s disease has not been consistently linked to visceral arterial aneurysm formation or to an increased rate of perioperative vascular complications. Therefore, the decision between endovascular intervention and open surgical repair should be based primarily on aneurysm anatomy, technical feasibility, and the ability to preserve hepatic perfusion [10]. Retrospective case series indicate that long‐term outcomes for visceral artery aneurysms are comparable after open or endovascular repair; however, morbidity is significantly higher with open repair compared to the endovascular approach [11]. Maintaining liver circulation is a crucial recommendation, regardless of procedure type, whether open or endovascular [1, 12]. Currently, while an endovascular‐first strategy is recommended for all HAAs when anatomically feasible, some authors suggest that surgical intervention may be warranted in cases of patient instability or for aneurysms exceeding 2 cm when endovascular approaches are not viable [13]. In the present case, surgical reconstruction of the vessel in order to preserve intrahepatic perfusion was considered the best technical solution for two primary reasons. First, the aneurysmatic involvement of the proper hepatic artery precluded the feasibility of embolization or ligation of the lesion due to the high risk of compromising hepatic perfusion from inadequate collateralization. Second, the placement of a covered stent was not feasible due to vessel tortuosity and the absence of a suitable distal neck for the aneurysm, which extended close to the proper hepatic bifurcation. Additionally, stenting would have required coverage of the gastroduodenal artery due to the specific anatomy, increasing the risk of hepatic ischemia in the event of acute stent thrombosis [14].Considering the relatively high morbidity associated with open repair, it is crucial to develop endovascular solutions for the minimally invasive resolution of potential surgical complications, as illustrated in our case with the successful embolization of left gastric artery bleeding, which was likely iatrogenic. Postoperative challenges such as bleeding and suspected anastomotic stenosis in our case must be interpreted in the correct clinical context. In diseases such as systemic lupus erythematosus or Behçet’s disease, vessel wall inflammation and immune‐mediated injury are well known to predispose to postoperative hemorrhage and graft complications. In contrast, Crohn’s disease is not typically associated with structural fragility of medium‐sized visceral arteries; vascular involvement is generally limited to microvascular ischemia or thromboembolic phenomena [15, 16]. In our patient, Crohn’s disease was in remission at the time of surgery, with no biochemical or clinical signs of systemic inflammation. We therefore attribute the postoperative bleeding from the left gastric artery to a likely iatrogenic event rather than to inflammatory fragility, while the transient suspicion of proximal anastomotic stenosis was not confirmed angiographically and probably reflected vessel remodeling. A limitation of this case is the lack of histological confirmation. However, the combination of radiological features, absence of systemic vasculitis or infection, and the patient’s comorbidities (metabolic syndrome, fatty liver disease, prior HBV infection without activity) made the diagnosis of a degenerative, atherosclerosis‐related true aneurysm the most plausible, and histology was therefore not pursued. At 12‐month follow‐up, the patient was asymptomatic with normal liver function tests; imaging confirmed graft patency with a stable, moderate, hemodynamically insignificant proximal anastomotic stenosis The previously described celiac trunk dissection persisted without progression or flow limitation. No new aneurysmal lesions or other complications were observed. In conclusion, this paper suggests that surgical repair could be a safe and effective option for HAAs when endovascular solutions are not feasible. Current practice involves individualized treatment decisions based on patient symptoms, comorbidities, and aneurysm location. Larger studies are needed to determine optimal management strategies.

## Conflicts of Interest

The authors declare no conflicts of interest.

## Funding

No funding was received for this manuscript.

## Data Availability

The data that support the findings of this study are available on request from the corresponding author. The data are not publicly available due to privacy or ethical restrictions.
